# Deciphering metabolic traits of the fungal pathogen *Aspergillus fumigatus*: redundancy vs. essentiality

**DOI:** 10.3389/fmicb.2012.00414

**Published:** 2012-12-03

**Authors:** Jorge Amich, Sven Krappmann

**Affiliations:** ^1^Research Center for Infectious Diseases, Julius-Maximilians-Universität WürzburgWürzburg, Germany; ^2^Microbiology Institute - Clinical Microbiology, Immunology and Hygiene, University Hospital of Erlangen, Friedrich-Alexander-Universität Erlangen-NürnbergErlangen, Germany

**Keywords:** aspergillosis, nutrients, gene family targeting, conditional promoter replacement, virulence

## Abstract

Incidence rates of infections caused by environmental opportunistic fungi have risen over recent decades. *Aspergillus *species have emerged as serious threat for the immunecompromised, and detailed knowledge about virulence-determining traits is crucial for drug target identification. As a prime saprobe, *A. fumigatus* has evolved to efficiently adapt to various stresses and to sustain nutritional supply by osmotrophy, which is characterized by extracellular substrate digestion followed by efficient uptake of breakdown products that are then fed into the fungal primary metabolism. These intrinsic metabolic features are believed to be related with its virulence ability. The plethora of genes that encode underlying effectors has hampered their in-depth analysis with respect to pathogenesis. Recent developments in *Aspergillus *molecular biology allow conditional gene expression or comprehensive targeting of gene families to cope with redundancy. Furthermore, identification of essential genes that are intrinsically connected to virulence opens accurate perspectives for novel targets in antifungal therapy.

## FUNGI AS OPPORTUNISTIC PATHOGENS

Pathogenic fungi have been classified historically in two major subgroups: primary and opportunistic ones (van Burik and Magee, 2001). While the former embraces fungi such as *Coccidioides immitis* or *Histoplasma capsulatum *that are capable of infecting a healthy host to cause severe systemic diseases, rely opportunistic fungi on an impaired host immune system to establish infection. Environmental saprobes, such as *Aspergillus fumigatus* or *Cryptococcus neoformans* are prominent examples from this group and rarely cause disease in a healthy individual. However, the number of associated clinical cases has significantly increased over the last decades, mainly based on modern medicine practices multiplying the number of immunosuppressed, susceptible individuals. Opportunistic fungi usually cannot thrive within the tissue of an immunocompetent individual. This suggests that they do not possess defined and unique characteristics to cause disease, features that have classically been coined as virulence factors (Casadevall and Pirofski, 1999). In fact, virulence of the two major fungal pathogens that are responsible for the majority of opportunistic infections, *Candida albicans* and *A. fumigatus*, appears to be multifactorial (Cutler, 1991; Latgé, 2001; Hube, 2004; Abad et al., 2010). This hampers the identification of specific targets against which chemotherapeutic compounds may be active. And indeed, current antifungal chemotherapy, which is mainly based on disrupting the integrity of the fungal cell membrane or wall (Hoehamer et al., 2010), is not satisfactory efficient.

It has become evident that nutritional versatility, uptake systems, and metabolic pathways employed by opportunistic fungi during infection represent fundamental aspects of their pathogenicity. Albeit considered as unspecific virulence determinants, such aspects may constitute novel targets for antifungal therapy. For instance, iron acquisition from host tissues has been proven to be important for virulence of several pathogens, among them fungi as* C. neoformans*, *C. albicans*, or *A. fumigatus* (Ramanan and Wang, 2000; Jung et al., 2008; Haas, 2012). In the latter, *in vivo* iron acquisition was shown to be dependent on the siderophore system and independent of the iron reductive assimilation pathway (Schrettl et al., 2004), establishing this process as a virulence attribute. This exemplifies how a deeper knowledge of the metabolic status and nutrient acquisition strategies within infected tissues sheds light on fungal virulence mechanisms to assist in identifying novel targets of antifungal therapy (Brock, 2009).

## THE HOST REPRESENTS A DISTINCT ENVIRONMENT DURING ASPERGILLOSIS

Invasive pulmonary aspergillosis (IPA) is a severe infection caused by *Aspergillus *species (Latgé, 1999) and affects primarily warm-blooded animals and as well as immunocompromised humans. Due to the success of immunosuppressive therapies in modern medicine has this disease had a steadily rising incidence during the last decades (McNeil et al., 2001). IPA is characterized by high mortality rates that may reach up to 90% depending on the host’s immune status (Singh and Paterson, 2005; Del Bono et al., 2008; Mikulska et al., 2009). Reasons for this are poor diagnostic means, the inefficiency of current antimycotic regimens (Baddley et al., 2010), and the general severness of the underlying diseases with their consequential risk factors. Among all *Aspergillus *species, *A. fumigatus* accounts for approximately 90% of all cases of aspergillosis (Brakhage and Langfelder, 2002). Publication of its genome sequence (Nierman et al., 2005) prompted researchers to look for relevant characteristics and specific genes that could explain this prominent virulence capacity. However, comparative genome analyses failed to identify such specific virulence factors (Tekaia and Latgé, 2005).

**FIGURE 1:**
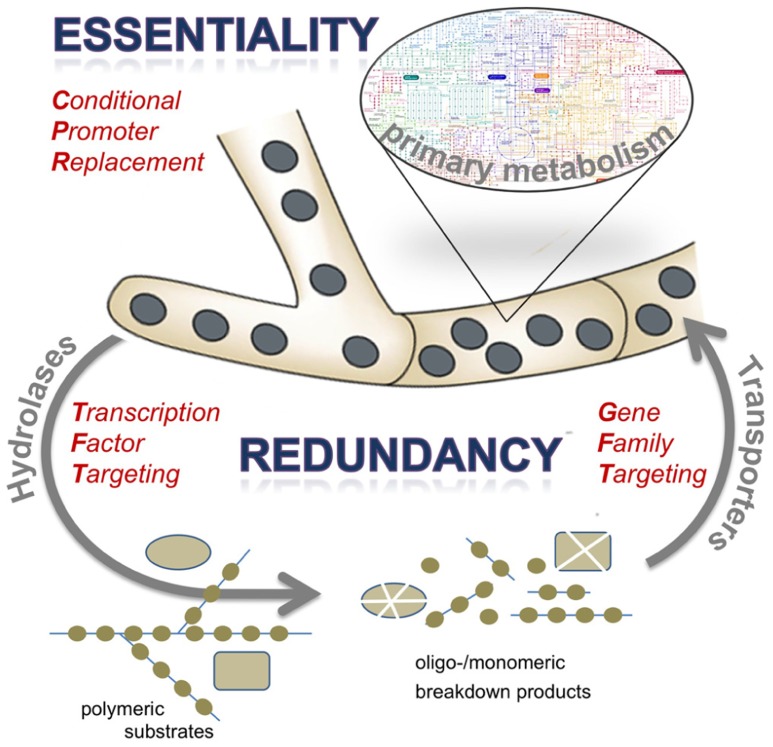
**Schematic presentation of the osmotrophic feeding style of the filamentous fungus *A. fumigatus* and consequences for antifungal target identification approaches.** Abundant secretion of extracellular hydrolases, especially proteases, results in degradation of complex substrates to break down their polymeric structure into oligomeric or monomeric subunits. These are taken up by numerous transport systems to be channeled in the primary metabolic routes of the pathogen. Both components of extracellular digestion and efficient uptake are characterized by a high degree of redundancy, whereas biosynthetic routes may harbor various gene products that are essential in the context of infection. Targets for antifungal therapy may therefore be identified by various experimental approaches, such as elimination of a regulatory protein acting on expression of extracellular enzymes (transcription factor targeting), deletion of a set of genes encoding redundant cellular activities (gene family targeting), or by conditional expression of candidate genes to test for essentiality (conditional promoter replacement). The latter approach has to be considered as most straight-forward and promising, given the vast redundancy among proteins expressed by *A. fumigatus* to support its saprobic lifestyle.

It has become more evident that its metabolic versatility enables *A. fumigatus* to thrive within the surrounding, infected tissues (Rhodes, 2006). In the course of pulmonary infection, *A. fumigatus *exploits the lung as principal metabolic environment and source of nutriments (**Figure 1**). In this respect, two major aspects are of special interest to understand the ability of this fungal pathogen to propagate in such a challenging environment: (i) the metabolic status of the fungus *in vivo* and regulatory cascades, metabolic processes, and stress responses that the fungus depends on within the surrounding tissue matrix, and (ii) the nutritional sources that the fungus exploits within such a highly specific growth medium, limiting nutrients, and the fungal anabolism. The following sections of this mini review will briefly summarize the current knowledge on nutrient acquisition and metabolic adaptation of *A. fumigatus* during invasive aspergillosis. Furthermore, it will outline approaches to overcome the negative consequences resulting from the pronounced redundancy of gene functions expressed by *A. fumigatus*, which complicates identification of novel antifungal drug targets.

## ADAPTING TO THE HOST

In its natural environment, the soil, *A. fumigatus* represents a prime saprobe that is characterized by its metabolic versatility and great adaptability. It has been suggested that such a competitive environment has served as evolutionary virulence school for this fungal pathogen (Askew, 2008; Fuller et al., 2011). This implies that *A. fumigatus* expresses distinct features supporting growth in human tissues based on adaptation to the adverse and variable conditions commonly encountered in the wild. Among these, its pronounced resistance to stressful conditions has to be taken into account. Reflecting elevated temperatures present in composting substrates, *A. fumigatus* is well adapted to temperature stress and displays an exquisite thermotolerance of up to 70°C (Bhabhra and Askew, 2005). Moreover, it is well adapted to grow at 37°C, the actual body temperature of humans, which represents an essential characteristic for pathogenicity. As a further example, hypoxia, which is commonly developed within compost piles (Wang et al., 2007), also develops during IPA in mice (Grahl et al., 2011) and, accordingly, *A. fumigatus*’ adaptation to hypoxic conditions is crucial for virulence (Willger et al., 2008).

In agreement with the importance of adaptation to the host, several stress response pathways and signal transduction cascades of *A. fumigatus* have been functionally characterized and their contributions to virulence were assessed (see review by Hartmann et al., 2011a). In essence, conditions of thermal stress, alkaline pH, hypoxia, iron depletion, and nitrogen starvation are encountered by the fungal pathogen when infecting a susceptible host. The ladder aspect became evidently clear from *in vivo* transcriptional profiling data that monitored the host adaptation process of *A. fumigatus* during early infection of neutropenic mice (McDonagh et al., 2008). By now, several additional profiling studies have sharpened our view on the transcriptional status of *A. fumigatus* under various environmental conditions that are relevant for pathogenesis, such as when feeding from proteinaceous substrates (Hartmann et al., 2011b), or when facing distinct stressors such as hypoxia or reactive oxidative species (McDonagh et al., 2008; Willger et al., 2008). The insights gained from such studies are instructive to identify cellular functions that are of relevance during infection and therefore assist in antifungal target identification. Validation of such promising candidates is commonly performed by approaches of reverse genetics: attenuated virulence of a corresponding *A. fumigatus* mutant still serves as most reliable and relevant phenotype in defining the fungal virulome for aspergillosis.

## TARGETING TRANSCRIPTIONAL REGULATORS

Transcription factors are fundamental for adapting to changing conditions by modifying gene expression patterns. Consequently, identification and elimination of *A. fumigatus* factors necessary to cope with the host environment are an obvious option to impair its virulence. For instance it has been reported that elimination of the *zafA* gene encoding a zinc response transcription factor renders *A. fumigatus* completely avirulent in a cortisone murine model of IPA (Moreno et al., 2007). Also, elimination of the HapX transcription factor, responsible for expression of iron-uptake related genes (Hortschansky et al., 2007), causes a significant reduction of virulence in neutropenic mice (Schrettl et al., 2010). Calcium signaling through the Ca^2^^+^-binding protein calmodulin constitutes one of the most promising candidates to develop new antifungal therapy (Steinbach et al., 2006; Steinbach et al., 2011). In line with this is the transcription factor CrzA, one key target of calcineurin signaling, fundamental for virulence in a neutropenic murine model of IPA (Cramer et al., 2008; Soriani et al., 2008). In addition, the *cpcA* gene, encoding the transcriptional effector of the cross-pathway control (CPC) system (also known as general control) of amino acid biosynthesis is expressed under amino acid-limiting conditions and was shown to contribute to *Aspergillus *virulence (Krappmann et al., 2004; Sasse et al., 2008). However, targeting transcription factors, despite of its potential positive perspective, might not always provide a comprehensive understanding of the metabolic *in vivo* status. Several examples exist where targeting the master regulator of a cellular process did not result in virulence attenuation: deleting the encoding gene of the transcriptional regulator YapA resulted in strikingly reduced resistance to oxidative stress but did not impair virulence (Lessing et al., 2007; Qiao et al., 2008); a deletant for the unique transcriptional regulator PrtT turned out to be severely impaired in extracellular proteolysis and unable to utilize proteinaceous substrates but its virulence was unaffected (Bergmann et al., 2009; Sharon et al., 2009). Negative results like this indicate that either the affected cellular function does not play any role for pathogenesis in the according disease model or that redundant factors take over from the deleted ones.

## COPING WITH GENETIC REDUNDANCY

Insights from recent studies on *A. fumigatus*’ virulence yielded the fundamental conclusion that this saprobe is well-equipped to sustain its nutritional supply in the host. However, the osmotrophic lifestyle of *Aspergillus*, i.e., extracellular digestion of complex substrates achieved by a plethora of secreted hydrolytic enzymes (Kobayashi et al., 2007; Monod et al., 2009) and followed by uptake of breakdown products via numerous transporters, impedes a comprehensive analysis (Yoon et al., 2009). To assist in assessing any relevance of cellular functions that are encoded by multiple genes, progress was made with respect to gene targeting approaches that allows repetitive targeting of all members of a gene family to abolish any redundantly encoded cellular function (d’Enfert, 1996; Krappmann and Braus, 2003; Krappmann et al., 2005; Nielsen et al., 2006; Forment et al., 2006; Hartmann et al., 2010). By developing genetic markers, recombinase-driven excision of which can be achieved *in fungo*, marker rescue became feasible to allow recurring rounds of gene targeting in a straight-forward manner. Depending on the number of genes to delete, such a gene family targeting approach might be worthwhile and instructive for identification of novel, fungal-specific virulence determinants. For instance, elimination of the eight-member *opt* gene family from the *A. fumigatus* genome was achieved in a pivotal study with the aim to investigate any supportive role of oligopeptide transport in pathogenesis (Hartmann et al., 2011b). The resulting octuple mutant did, however, not display any differences in virulence in comparison to its wild-type progenitor. Even interfering with extracellular proteolysis of this strain by additionally deleting the *prtT* gene did not alter its virulence capacities, again highlighting the pronounced degree of redundancy encoded by the *A. fumigatus* genome. However, this study proves that recyclable marker systems nowadays allow for straight-forward repetitive gene targeting to assist in elucidating redundant features of *A. fumigatus*. This approach facilitates identification of relevant antifungal drug targets that are based on gene family products.

## BIOSYNTHETIC ROUTES AND ESSENTIAL GENES AS PRIMARY TARGETS

The metabolic versatility displayed by *A. fumigatus* supports its growth and therefore virulence inside a susceptible host (Paisley et al., 2005). As described above, detailed insights in the metabolic situation was gained by *in vivo* transcriptional profiling studies, indicating that a pronounced starvation for nitrogen reflects the nutritional status of *A. fumigatus* when germinating in the lungs of neutropenic mice (McDonagh et al., 2008). In line with this, several gene products involved in nitrogen sensing and utilization had been characterized before to influence *A. fumigatus *virulence (reviewed by Krappmann and Braus, 2005). With respect to carbon, detailed analysis of a mutant strain impaired in methylcitrate cycling suggested that proteins may serve as primary source of this macroelement, a finding that is in accordance with the reported full virulence of a mutant compromised in fatty acid metabolism (Schöbel et al., 2007; Ibrahim-Granet et al., 2008). Yet, specific nutritional sources during IPA remain unidentified so far.

Despite this ignorance, several anabolic genes with their respective products have been identified to be essential for growth during pulmonary aspergillosis. In a seminal study, biosynthesis of the folate precursor *para*-aminobenzoic acid had been demonstrated to be strictly required for *A. fumigatus* pathogenicity in several disease models and still serves as a paradigm for an anabolic route being essential for pathogenesis (Brown et al., 2000; Slater et al., 2011). By further mutant analysis, several biosynthetic pathways had been identified to support fungal growth in the nutritionally scarce environment of murine lung tissue. One best known example is uracil biosynthesis represented the *pyrG* gene, which encodes orotidine-5′-phosphate decarboxylase to catalyze the final step of *de novo* UMP biosynthesis. Mutants deleted for the *pyrG* gene are auxotrophs for uracil/uridine and display a severe reduction in virulence when tested in a non-neutropenic model of murine invasive aspergillosis (d’Enfert et al., 1996). As a strictly fungal-specific metabolic pathway, lysine biosynthesis turned out to be essential for full virulence, validated by infections with defined mutants deleted either for the *lysF* or the *hcsA* gene (Liebmann et al., 2004; Schöbel et al., 2010). Furthermore, essentiality of the siderophore biosynthesis pathway was demonstrated by elimination of the gene *sidA* encoding L-ornithine-*N*^5^-monooxygenase, which catalyzes the first committed step of hydroxamate-type siderophore biosynthesis (Schrettl et al., 2004).

In a further development of deleting biosynthetic genes to generate auxotroph strains, pathways that are required for growth under any condition tested, and that are therefore generally essential, represent an attractive option to be selected as targets for antifungal therapy. Since the functions of the corresponding proteins are fundamental for fungal growth, their impairment would consequently eliminate virulence. An elegant approach for essential gene identification was validated by Romero et al. (2003) who developed a conditional promoter replacement (CPR) strategy further by employing the nitrogen-regulated *A. fumigatus nii A* promoter (p*NiiA*; Romero et al., 2003; Hu et al., 2007). In this setup, expression of the gene of interest is blocked in the presence of ammonium, whereas nitrate as N-source allows proper expression of the gene under p*NiiA* control. The power of this system was demonstrated by the close phenotypic correlation between p*NiiA*-CPR strains under repressing conditions with respective disruption mutants. Furthermore, such conditional strains could be directly tested for virulence in animal models, although being limited to systemic infections. In fact, 35 essential genes out of an initial screening pool comprising 54 candidates were identified in this seminal study, which may now be subjected to further analysis. Therefore, the CPR approach represents a valid and most straight-forward method for large scale essential gene identification.

## NOVEL TOOLS PROVIDE PROMISING PERSPECTIVES

The most promising strategy for drug target prioritization to fight aspergillosis lies in the identification of non-redundant and essential gene products. As future trend emerging from distinct candidate approaches, comprehensive, large scale screening studies are reasonably required. These could include the generation of a collection of defined deletion mutants, comprising every single gene annotated from a refined *A. fumigatus* genome sequence. Including conditional alleles would further scrutinize essential cellular functions that are based on single gene products and appears as a superior strategy. In particular, recent developments that demonstrated the feasibility of doxycycline-dependent expression modules in *Aspergillus* (Vogt et al., 2005; Meyer et al., 2011; Dichtl et al., 2012) pave the road for such advanced CPR studies that might even allow for virulence tests in pulmonary aspergillosis models. As an alternative to extensive and randomized screening, network approaches of systems biology might serve as more efficient and straight-forward tactic (Chavali et al., 2012; Horn et al., 2012). In conjunction, new molecular tools for multiple gene targeting will allow comprehensive understanding of the *in vivo* *A. fumigatus* metabolic status. Concluding, the advanced molecular biology of *A. fumigatus *(Krappmann, 2006) holds the future promise of innovative strategies for antifungal drug target identification, an urgent task in fighting against this first mould pathogen in Europe (Senior, 2009).

## Conflict of Interest Statement

The authors declare that the research was conducted in the absence of any commercial or financial relationships that could be construed as a potential conflict of interest.
